# COVID Lockdowns, Social Distancing, and Fatal Car Crashes: More Deaths on Hobbesian Highways?

**DOI:** 10.1007/s41887-020-00059-8

**Published:** 2020-12-21

**Authors:** Marshall W. Meyer

**Affiliations:** Wharton School, University of Pennsylvania, Philadelphia, PA USA

**Keywords:** COVID, Social distancing, Fatal vehicle crashes, Hobbesian theory, Compliance, Social norms, Traffic laws

## Abstract

**Research Question:**

What happened to US traffic safety during the first US COVID-19 lockdown, and why was the pattern the opposite of that observed in previous sudden declines of traffic volume?

**Data:**

National and local statistics on US traffic volume, traffic fatalities, injury accidents, speeding violations, running of stop signs, and other indicators of vehicular driving behavior, both in 2020 and in previous US economic recessions affecting the volume of road traffic.

**Methods:**

Comparative analysis of the similarities and differences between the data for the COVID-19 lockdown in parts of the USA in March 2020 and similar data for the 2008–2009 global economic crisis, as well as other US cases of major reductions in traffic volume.

**Findings:**

The volume of traffic contracted sharply once a COVID-19 national emergency was declared and most states issued stay-at-home orders, but motor vehicle fatality rates, injury accidents, and speeding violations went up, and remained elevated even as traffic began returning toward normal. This pattern does not fit post-World War II recessions where fatality rates declined with the volume of traffic nor does the 2020 pattern match the pattern during World War II when traffic dropped substantially with little change in motor vehicle fatality rates.

**Conclusions:**

The findings are consistent with a theory of social distancing on highways undermining compliance with social norms, a social cost of COVID which, if not corrected, poses potential long-term increases in non-compliance and dangerous driving.

## Introduction

A 20-year-old man was killed and a woman was injured in a crash early Friday on the Benjamin Franklin Parkway, Philadelphia police said. The pair were among four people in a black Volkswagen Jetta that investigators believe was speeding up the Parkway about 4:20 a.m. when the driver apparently lost control of the car near 20th Street, then struck a tree and a pole on the center median. An eyewitness told police the car was one of three driving fast at the same time, but Capt. Mark Overwise of the Accident Investigation District said it didn’t appear to be a drag race or “drag racing the way you see it in South Philly,” with a start and finish line. (Shaw 2020)

Something unusual happened beginning in March 2020. While driving decreased substantially as COVID-19 spread and stay-at-home orders were issued in late March and early April, motor vehicle fatality rates and the severity of accidents shot up for the cars remaining on the road. There is almost no precedent for a simultaneous decline in traffic and spike in fatality rates and accident severity. In past economic downturns, declining traffic has led to declines in fatality rates and accident severity. A partial explanation, I’ll argue, lies in the suddenness and magnitude of the decline that created an experiment in social distancing, in this instance between drivers, unlike any within memory and with consequences neither our politicians nor our public health experts fully anticipated though some traffic engineers did.

My argument is that, in the time of COVID-19, social distancing in the context of an inchoate government response has contributed to the fraying of social relations and social norms. Though intended to limit the transmission of COVID-19, social distancing has had substantial and observable social costs, and these costs may be enduring.

I begin from a sociological perspective. The highways are a microcosm of society, and what we are sensing in society is more readily observable on the highways. Here I will connect fatalities and injuries on the highways with our management, or lack of management, of our response to COVID-19 and how it has disrupted society. I argue that our highways have become Hobbesian to draw attention to and provide a quantitative glimpse into larger problems triggered by our response to COVID-19.

I’ll begin with a brief exposition of Hobbes’s *Leviathan*. I’ll then argue that we have performed an experiment in social distancing on the highways and this experiment seems to have made driving more like Hobbes’s “state of nature”—in other words, the rules of the road have gone into abeyance and driving has become more dangerous. This will be backed by data on traffic volume, fatalities, injury accidents, speeding violations, and even the running of stop signs. These data show essentially two things: first, the volume of traffic contracted sharply once a COVID-19 national emergency was declared and most states issued stay-at-home orders; and, second, motor vehicle fatality rates, injury accidents, and speeding violations went up, in some instances sharply, and remained elevated even as traffic began returning toward normal. This pattern does not fit post-World War II recessions where fatality rates declined with the volume of traffic nor does the pattern fit World War II when traffic dropped substantially with little change in motor vehicle fatality rates.

I’ll then seek to explain why the roads and highways have become more dangerous since COVID-19. A simple model from traffic engineering helps account for Hobbesian highways in the aftermath of stay-at-home and business shutdown orders. It also helps explain why motor vehicle fatality rates have dipped in past economic recessions. But it does not explain why motor vehicle *fatality rates have remained high as the USA reopened*. I borrow a threshold model from studies of small groups, innovation, and the sociology of collective behavior—the behavior of crowds and mobs—to explain this. I then return to the larger theme that the COVID-19 pandemic and social distancing eroded many critical though invisible social norms including everyday norms of driving on the highways as the highways emptied of traffic. I’ll end with a mixed message. We have addressed highway safety before and can do it again. Still, the *social* costs of COVID-19, among them Hobbesian highways, need attention.

## Hobbes in Brief

No lengthy exposition of Thomas Hobbes’s *Leviathan* is needed. The key point is that the Hobbesian world lacks a social fabric. There are two possible conditions, an anarchic state of nature and a commonwealth whose citizens are subjugated to a sovereign. In the state of nature, there are no enduring social bonds or obligations. In the commonwealth, citizens’ sole obligation is to the sovereign.

The first and most famous of the two conditions is Hobbes’s state of nature or “naturall condition of mankind” where passions are unchecked and conflict is rampant. “There Is Alwayes Warre Of Every One Against Every One. . . And the life of man, solitary, poore, nasty, brutish, and short” (Hobbes, 1651 [Hobbes 2009], p. 212). Anything goes. Force and fraud are acceptable, and “there be no Propriety, no Dominion, no Mine and Thine distinct; but onely that to be every mans that he can get; and for so long, as he can keep it” (p. 214). Interestingly, agreements made under duress remain valid. “Covenants entred into by fear, in the condition of meer Nature, are obligatory. For example, if I Covenant to pay a ransome, or service for life, to an enemy; I am bound by it” (p. 240).

Hobbes’s state of nature is largely a rhetorical device intended to grab attention. The state of nature did not exist in the seventeenth century and does not exist today. Still, the state of nature has become all but synonymous with Hobbes. It is worrisome, and it is the specter used by Hobbes to justify absolute authority just as it is by politicians currently embracing “law and order.”

Here is the argument for absolutism: the first and fundamental law of nature is “To seek Peace, and follow it” (p. 221). However, so long as “this naturall Right of every man to every thing endureth, there can be no security to any man. .. of living out the time” (p. 220). Hence, all must agree whether voluntarily or by force to renounce most of their rights and transfer them irrevocably to a sovereign with sole power to maintain the peace. Hobbes’s alternative to the Hobbesian state of nature, then, is absolutism, and philosophers, political scientists, and sociologists since have struggled to find less draconian solutions to the problem of order posed by Hobbes. How we will address the same problem of order on our highways, should they remain persistently Hobbesian as traffic returns toward normal volume, is uncertain. Should motor vehicle fatality and injury rates not return to normal, which they have not so far, we may have to consider whether a regime of strict enforcement is necessary and politically acceptable.

## Social Distancing and Highway Safety

Few think of roads and highways as arenas for social behavior. Rather, vehicles are treated as physical objects occupying physical space, and the greater the spacing between vehicles the less likely they are to collide. Normally, the model of vehicles as physical objects in physical space holds since the rules of the road, especially the unwritten rules learned from experience, change slowly if at all. The question here is whether under some circumstances increased physical distance between vehicles can also cause drivers to become inattentive to each other and to the norms of driving they otherwise respect—in other words, can physical distancing of vehicles lead to social distancing of drivers with consequences opposite to those expected from physical distancing? The intuition is that a sharp and unexpected increase in the physical distancing of vehicles can create a perception that it is safe to abandon lifelong driving habits. Somewhat differently and more sociologically, if increases in dangerous driving, injury accidents, and vehicle fatalities follow sudden and substantial decreases in traffic flow, then might a reasonable inference be that that physical distancing of vehicles has also socially distanced their drivers?

### US Vehicular Traffic in 2020

To begin, I’ll describe vehicular traffic in the USA in the spring and summer of 2020. Forty-two of the 51 states and the District of Columbia announced stay-at-home orders from March 19 (California) to April 6 (Missouri). All states except Nebraska, Wyoming, and South Dakota closed bars, restaurants, and non-essential retailers early in the COVID-19 pandemic. The impact of these stay-at-home and shutdown orders on vehicular traffic was wholly without precedent in the USA. Exhibit 1 shows the millions of vehicle miles traveled monthly in the USA from January 1970 to August 2020 reported by the Federal Highway Administration. The data are not seasonally or otherwise adjusted; hence, vehicle miles peak in the summer, almost always in July, and bottom out in mid-winter, usually February. The gray vertical bars in Exhibit 1 are periods of economic recession. As can be seen in Exhibit 1, vehicle miles trend steadily upward from 1970 through 2019 except during recessions and, in the case of the 2008–2009 recession, for several years beyond. The experience of 2020 following COVID-19 was wholly different, however. Traffic dropped 19% in March 2020 compared to March 2019 and plummeted 40%, from 282 to 168 billion vehicle miles, in April 2020 compared to April 2019. To put this in perspective, COVID-19 shutdowns reduced US vehicular traffic in April 2020 to the volume seen 32 years before in April 1988. Nationwide traffic volumes recovered somewhat in May and June and peaked in July at 262 billion vehicle miles, declining 26%, 13%, and 11% respectively from 2019. However, motor vehicle travel then slipped somewhat to 251 billion vehicle miles in August, a 12% decline from 2019.

**Exhibit 1:**
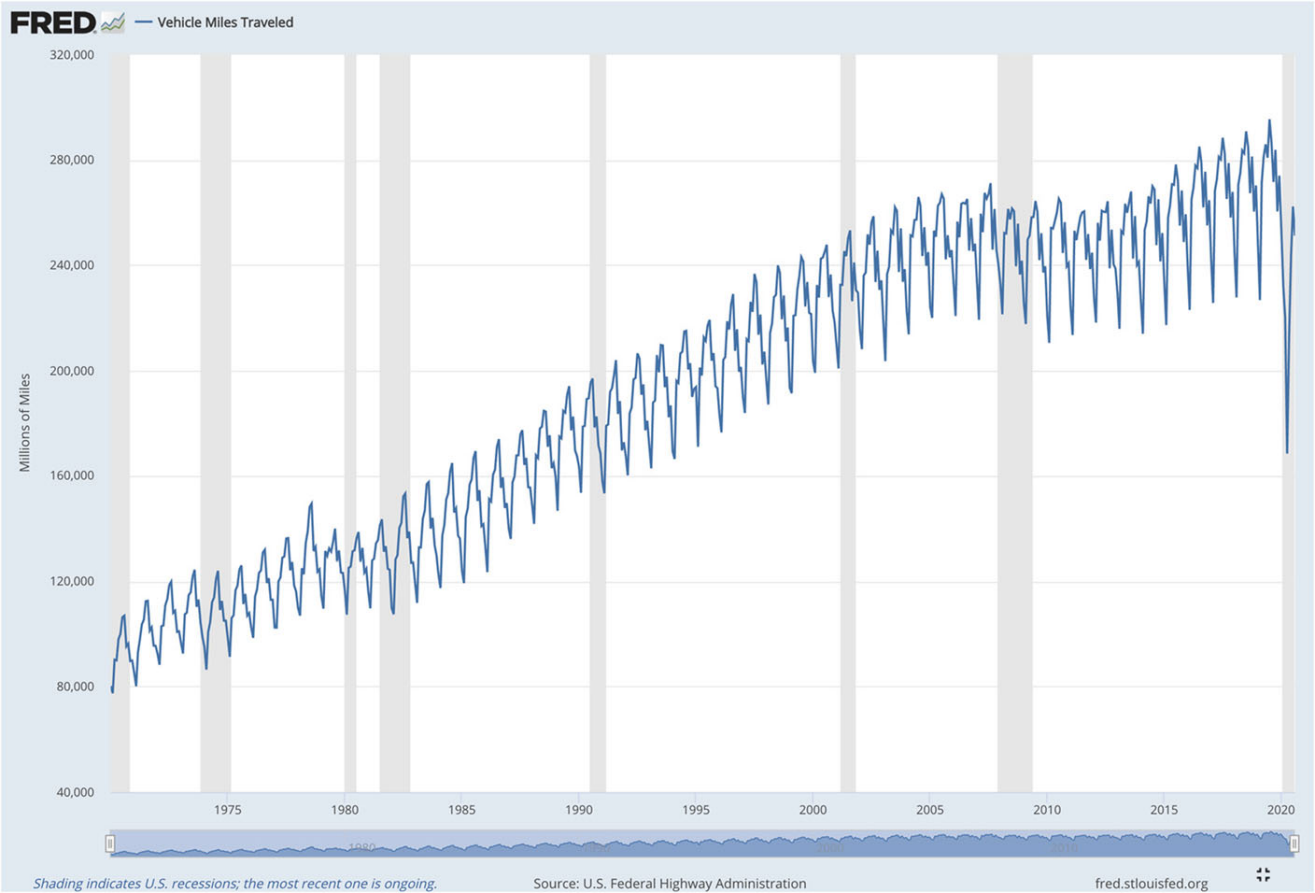
US millions of vehicle miles traveled by month, January 1970–August 2020. Source: U.S. Federal Highway Administration 2020c

By comparison, the sharpest drop in US vehicle miles from World War II to 2019 occurred in the 2 years before the Great Recession, 2006–2008. The decline of traffic, over 2 years, was 2.6%, which a Brookings Institution report called “historic” (Puentes and Tomer 2008). The truly historic decline in US vehicle miles, in fact, occurred when passenger car gasoline rationing was mandated following the US entry into World War II. Automotive vehicle miles in 1942 were 21% lower than in 1941 and dropped another 26% in 1943, a 43% decline from the 1941 peak. Truck traffic also dropped but not as sharply, declining 15% in 1942 and another 9% in 1943 (Davis 2020).

Some of the most current state-level traffic data are published by the Commonwealth of Massachusetts. Massachusetts monitors vehicle flows at 39 representative stations and reports these flows and other key traffic statistics weekly. Exhibit 2 displays 2019 and 2020 weekly average traffic flows as well as the percentage change in these flows for all 39 stations from January through September. As can be seen, traffic volume dropped by more than half in early April 2020 and recovered to 78% of 2019 levels by mid-September 2020.

**Exhibit 2:**
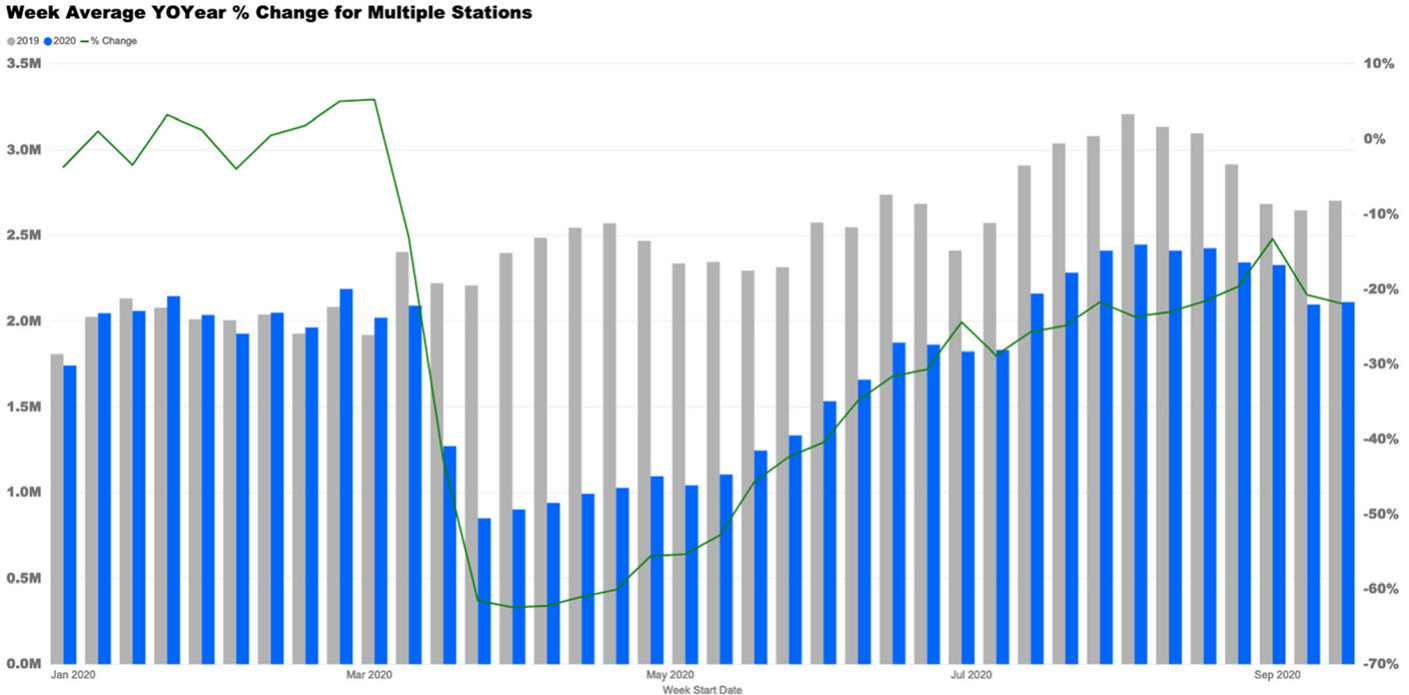
Massachusetts traffic volumes at representative locations January–September 2019–2020. Source: Commonwealth of Massachusetts 2020

Early April declines in traffic volumes exceeding 50% were also reported in several US major metropolitan areas:

In one measure of declining road use, the average number of miles traveled in 24 hours by vehicles in the New York metro region, which includes parts of New Jersey and Connecticut, had plunged 64 percent by April 2, according to INRIX, a transportation analytics company. It was the largest drop in the nation, followed by Detroit, which fell 62 percent, and San Francisco, 60 percent. Los Angeles, another famously traffic-choked city, dropped 53 percent (Hu 2020).

Rural motor vehicle traffic in the US declined in tandem with urban traffic beginning in March 2020, though by somewhat smaller percentages. Exhibit 3 shows the *ratio* of 2020 to 2019 motor vehicle miles traveled on urban and rural roadways from January through March—the lower the ratio, the greater the year-on-year decline in traffic. Urban mileage bottomed in April 2020 at 59% of April 2019 urban mileage, while rural mileage in April 2020 was 63% of 2019 rural mileage. By August, urban vehicle miles traveled returned to 86% of 2019 mileage, while rural mileage returned to 90% of 2019 mileage.

Save for World War II—more on World War II shortly—there is no US precedent for the swiftness of the reduction in motor vehicle miles occurring in late March and April 2020 and the magnitude of this reduction. The thinning of traffic was due to stay-at-home orders and business shutdowns aimed at containing COVID-19. It was especially marked in major metropolitan areas and slightly attenuated in rural areas of the USA. The consequences of this emptying of US roads and highways were profound.

### Motor Vehicle Fatality Rates

I’ll begin with nationwide motor vehicle fatality rates compiled by the National Safety Council following COVID-19. The NSC and almost all other safety advocates calculate rates as the ratio of deaths caused by motor vehicles to the number of miles driven. Motor vehicle fatality rates gauge the risk of driving, that is, your risk of dying or causing others including pedestrians to die *when you are on roads and highways*. It is not the risk to the entire population of dying in a motor vehicle accident.^1^ The conventional metric is motor vehicle fatalities per 100 million vehicle miles. Common sense would suggest that the fewer vehicles on the road, as has happened since COVID-19, the fewer the opportunities for collisions and hence, the less the risks of driving and the fewer fatalities per mile driven. This has almost always been the case in the past. Following stay-at-home orders and business shutdowns ordered early in the COVID-19 pandemic, however, US motor vehicle fatality rates increased significantly.

Here are three monotonously similar press releases from the National Safety Council reporting motor vehicle fatality rates in March, April, and May 2020:

#### Motor Vehicle Fatality Rates Jump 14% in March Despite Quarantines

May 20, 2020

Itasca, IL – Preliminary estimates from the National Safety Council show that as Americans began driving less and covering fewer miles, the emptier roads became more lethal. Early data indicate a year-over-year 14% jump in fatality rates per miles driven in March, in spite of an 8% drop in the total number of roadway deaths compared to March 2019. The actual number of miles driven dropped 18.6% compared to the same time period last year. The mileage death rate per 100 million vehicle miles driven was 1.22 in March compared to 1.07 in March 2019 (National Safety Council 2020a).

#### Motor Vehicle Fatality Rates Jump 36.6% in April Despite Quarantines

June 23, 2020

Itasca, IL – Preliminary estimates based on April data from all 50 states indicate that for the second straight month, Americans did not reap any safety benefit from having less roadway traffic. In fact, the roads became even more lethal as miles driven plummeted. Preliminary estimates from the National Safety Council show a year-over-year 36.6% jump in fatality rates per miles driven in April, in spite of an 18% drop in the total number of roadway deaths compared to April 2019. The actual number of miles driven dropped 40% compared to the same time period last year. The mileage death rate per 100 million vehicle miles driven was 1.47 in April compared to 1.08 in 2019 (National Safety Council 2020b).

#### Motor Vehicle Fatality Rates Rose 23.5% in May, Despite Quarantines

July 21, 2020

Itasca, IL – Preliminary estimates from the National Safety Council based on May data from all 50 states indicate that for the third month in a row, road users in the USA were at a higher risk of dying from a motor vehicle crash. As reported in *Injury Facts*, the fatality rate per miles driven in May—when most of the country was deep in quarantine from the pandemic—jumped a staggering 23.5% compared to the previous year, despite far less traffic on the roads. The number of miles driven in May dropped 25.5% compared to the year prior. The increased rate comes in spite of an estimated 8% drop in the number of deaths for May compared to the prior year. Overall, the mileage death rate per 100 million vehicle miles driven was 1.47 in May compared to 1.19 in 2019 (National Safety Council 2020c).

The term “preliminary estimates” requires explanation. National Safety Council fatality statistics are dynamic. They are updated continuously as deaths occurring within a year of vehicular accidents are recorded. NSC’s estimates of March, April, and May 2020 motor vehicle fatality rates as of this writing, November 2, 2020, are slightly higher than the preliminary estimates above, 1.24, 1.48, and 1.56 deaths per hundred million miles in the three months, respectively. In the subsequent discussion, I will use NSC’s November 2 estimates of 2020 monthly motor vehicle fatality rates rather than the preliminary estimates in their press releases above.

There was some good news in March, April, and May 2020: year-on-year *numbers* of motor vehicle fatalities were down (from 2910 to 2710 or 7% in March, from 3040 to 2490 or 18% in April, and from 3410 to 3280 or 4% in May). However, the number of miles driven declined faster (by 19% in March, 40% in April, and 26% in May) so that the motor vehicle fatality rate increased over the same month in 2019 by 15% in March, 37% in April, and 29% in May. June 2020, however, shattered the March–May pattern. Nationwide, the *number* of motor vehicle fatalities in June 2020 was 3990 compared to 3420 in 2019, up 17%. The 17% increase in fatalities combined with a 13% decrease in vehicle miles driven yielded a 34% increase in the motor vehicle fatality rate, from 1.22 in June 2019 to 1.64 in June 2020. July basically continued June with 4020 estimated motor vehicle fatalities compared to 3530 in July 2019, up 14%. The 14% growth in fatalities, combined with an 11% decrease in vehicle miles driven, yielded a 27% increase in the July motor vehicle fatality rate, from 1.20 per 100 million miles driven in 2019 to 1.53 in 2020. And August was not dramatically different from July with 4010 estimated motor vehicle fatalities compared to 3570 in August 2019, up 12%, vehicle miles down 13%, and the motor vehicle fatality rate up 29% from 1.24 to 1.60 per 100 million vehicles miles. July 2020 also marked an unfortunate milestone as it was the first month since July 2007 when motor vehicle fatalities exceeded 4000 (National Safety Council 2020e).

Exhibit 3 displays how unusual the relationship of vehicular travel to fatality rates became once COVID-19 struck the USA. The vertical bars in Exhibit 4 are monthly vehicle miles traveled in millions, and the orange line is the monthly motor vehicle fatality rate, both shown from January 2015 to August 2020. Aside from seasonal variation, vehicular travel tended slightly upward while fatality rates remained essentially flat from January 2015 through February 2020. Beginning in March 2020, however, a jaw opened as traffic plummeted and fatality rates shot up. The jaw closed slightly in July and opened again in August, though by how much will not be known until August motor vehicle fatalities are fully reported.

**Exhibit 3:**
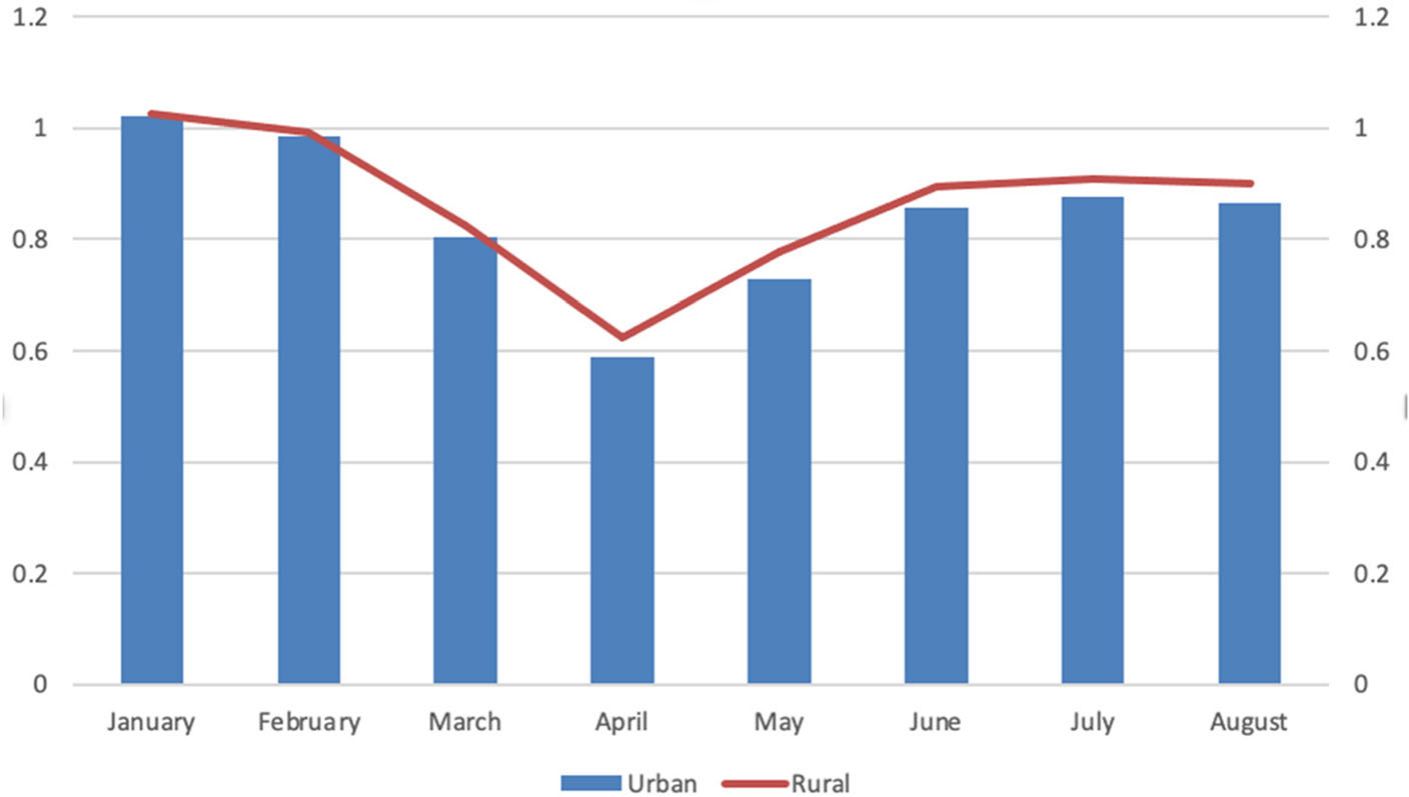
Ratio of 2020 to 2019 vehicle miles traveled on urban and rural roadways, January–August. Source: U.S. Federal Highway Administration 2020a

**Exhibit 4:**
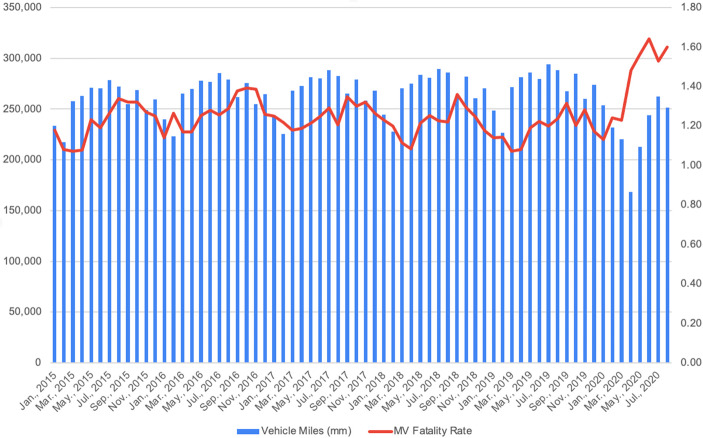
Monthly vehicle miles traveled and motor vehicle fatality rates. January 2015–August 2020. Source: National Safety Council 2020e

Motor vehicle fatality rates in major metropolitan areas appear to have paralleled national trends. In New York City, January 1–September 27, 2020, motor vehicle fatalities increased 2.5%—from 163 to 167—compared to the same dates in 2019 despite the dramatic plunge in metropolitan area traffic volume from early April (New York City Police Department 2020b). Los Angeles motor vehicle fatalities were flat through mid-May despite a similar COVID-19 induced drop in traffic. On May 14, the Los Angeles Times reported:

So far this year, 86 people have been killed in traffic collisions on city streets, Commander Marc Reina of the LAPD’s Traffic Group said at a news conference Thursday. “To put that in perspective, year-to-date, 89 people have been the victim of homicide within the city of Los Angeles,” Reina said. “Even with the stay-at-home orders still in effect, we’re currently at the same amount of [traffic] fatalities that we had at this time last year” (Fonseca 2020).

Half of these January–May 2020 Los Angeles motor vehicle fatalities were pedestrians.

### Speeding Violations and Injury Accidents

The same pattern observed for motor vehicle fatalities post-COV1D-19 appears in data on speeding violations and injury accidents. Some of most complete data are from New York City, which though hardly representative of the USA is advantageous because NYPD traffic statistics are released promptly and allow us to see moving violations and collisions before and after COVID cases peaked in New York (the peak number of new COVID-19 diagnoses was 11,571 on April 14). Exhibit 5 displays NYPD reports of moving violations and vehicle collisions for March through August 2019 and 2020. The upper panel of the table shows 2019 statistics and the lower panel 2020.

**Exhibit 5:**
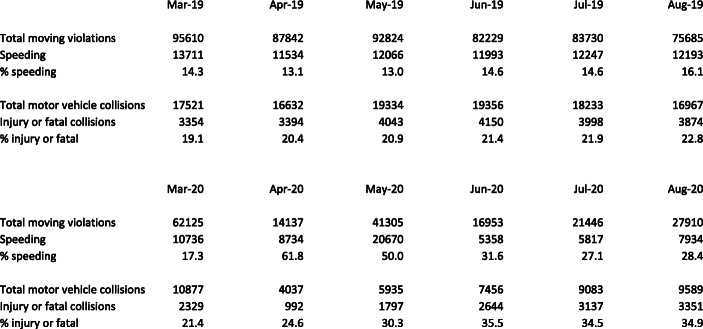
NYPD citywide moving violations and vehicle collisions March–August 2019 and 2020. Source: New York City Police Department 2020a

Let us first compare moving violations in 2019 and 2020. The drop-off is dramatic from April onward. April moving violations plunged from 87,842 in 2019 to 14,137 in 2020, May from 92,824 to 41,305, June from 82,229 to 16,953, July from 83,730 to 21,146, and August from 75,685 to 27,910. Next, let us look at the percentage of violations for speeding. Speeding citations as a percentage of moving violations were nearly flat from March to July 2019, 13 to 16%, and were 17% of moving violations in March 2020. However, in April 2020, when the number of traffic citations dropped by more than three-quarters, 62% of violations in New York City were for speeding. The percentage of speeding violations then declined to 50% in May, 32% in June, 27% in July, and 28% in August, 2020, still 75% higher than in August 2019.

The severity of New York City motor vehicle collisions also changed remarkably. There is a dramatic drop-off in April collisions, from 16,632 in 2019 to 4037 in 2020, followed by a steady increase to 5935 in May, 7456 in June, 9083 in July, and 9589 in August 2020. The percentage of collisions involving injuries or fatalities remained almost flat, from 19 to 23%, from March to August 2019 and was 21% in March 2020. However, that percentage—of injury and fatal accidents—increased to 25% in April, 30% in May, 36% in June, and 35% in July and August 2020. While the trends in New York City speeding violations (as a percentage of traffic citations) and injury and fatal accidents (as a percentage of all accidents) from April to August 2020 do not coincide, both were substantially higher in than in 2019. By these measures, driving in New York City became more hazardous in the time of COVID-19.

The ratio of citations to accidents provides a rough gauge of the intensity of traffic enforcement in New York City. A comparison of August 2019 with August 2020 could be the most apt. In August 2020, the NYPD wrote 27,910 citations for moving violations compared to 75,685 citations in August 2019 or 63% fewer. The number of accidents decreased from 16,967 to 9589 or 39% while the number of injury and fatal accidents decreased from 3874 to 3351 or 13% from August 2019 to August 2020. While these comparisons are imperfect, they are consistent with reduced intensity of traffic enforcement.

We can also compare moving violations in the Commonwealth of Massachusetts for the months of January–August in 2019 and 2020, shown in Exhibit 6. The Massachusetts rate of speeding violations (as a percentage of total citations) remained near 30% from January to August 2019 as well as from January to March 2020. The percentage of speeding violations rose to 35% in April and 52% in May after which it declined to 47% in June, 38% in July, and returned to 35% in August. Most striking in Massachusetts and not reported for other jurisdictions to the best of my knowledge are citations for speeding in excess of 100 miles per hour, a misdemeanor offense. Massachusetts monthly citations for speeding in excess of 100 MPH remained well under 200 through April 2020. In May, however, they skyrocketed to 380 and subsequently fell back to 245 in both June and in July and then to 211 in August. It is hard to interpret the Massachusetts’ total number of traffic citations since the numbers are extremely high in January and February 2019 compared to subsequent months. Notable, however, is that August 2020 traffic citations, 46,545, are within 1% of the August 2019 tally, 47,078.

**Exhibit 6:**
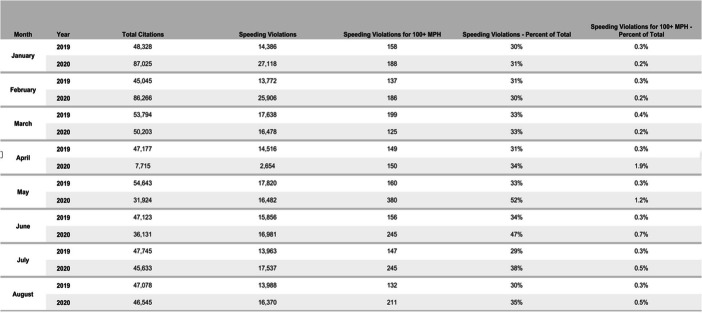
Massachusetts traffic violations, speeding violations, and speeding violations in excess of 100 MPH. January–August 2019 and 2020. Source: Commonwealth of Massachusetts 2020

### Truckers’ Behavior

According to Teletrac Navman (2020), the mileage driven by commercial vehicles dropped 20% in the 5 weeks from March 13, when the USA declared a national COVID-19 emergency, through April 17. In the same interval, mileage driven above the posted speed limit by vehicles tracked by Teletrac Navman increased 17%, harsh cornering events increased 15%, and failures to stop at stop signs increased 10%. These percentages reflect changes in the actual number of events among the commercial vehicles tracked by Teletrac Navman. Most interesting, perhaps, is that harsh braking events decreased by 54%, despite increases in speeding, running stop signs, and high-speed cornering, suggesting that the sparseness of traffic-induced truck drivers to take more risks on the road.

### Summary

A sharp decrease in traffic volume coupled with noticeable increases in motor vehicle fatality rates, proportions of injury and fatal accidents, speeding tickets in proportion to traffic citations, and in Massachusetts speeding in excess of 100 MPH occurred in the wake of COVID-19 stay-at-home orders.

These observations seem anomalous requiring an unusual explanation. The principal hypothesis is that sudden physical distancing of vehicles exacerbated social distance among drivers and, as a consequence, norms and habits of driving learned over decades fell into desuetude. A secondary hypothesis is that fear of COVID-19 infection caused police officers to keep distance from motorists and, as a consequence, to diminish traffic enforcement efforts. I cannot prove either of these hypotheses from the data at hand. The Teletrac Navman trucking data (increased speeding, harsh cornering events, and running stop signs but fewer harsh braking events) though limited are highly suggestive regarding the first. The disproportionate decline of total traffic citations in New York City and Massachusetts are suggestive of the second. Neither drivers’ distancing from each other and disregard of the rules nor the distancing of police officers from drivers, both the consequence policies taken or not taken to respond to COVID-19, can be considered favorable outcomes. I will now show how unfavorable these outcomes have been in comparison with impact of periodic recessions and of wartime driving restrictions.

## Historical Motor Vehicle Fatality Rates

Historical data show that COVlD-19’s impact on highway safety has been highly unusual. Exactly how unusual depends on interpretation of driving data from World War II. First, motor vehicle fatalities and fatality rates declined during eight of the ten recessions occurring from 1950 to 2018, in sharp contrast to increased fatality rates in the aftermath of COVID-19. Next, motor vehicle fatality rates in the 50 states declined significantly with unemployment in the 11 years during and adjacent to the Great Recession, 2003 to 2013, and the unemployment rate was associated with reduced vehicle mileage in 88 Ohio counties from 2009 to 2013. Again, these results are inconsistent with what we have recently experienced. Finally, motor vehicle fatality rates during World War II, when the government suppressed driving, remained essentially flat, which seems to differ from the post-COVID-19 experience. An open question is whether gasoline rationing, a 35 mile-per-hour national speed limit, and government campaign against unnecessary driving, other things being equal, should have driven motor vehicle fatality rates sharply downward, which they did not.

### Motor Vehicle Fatalities and Fatality Rates Since World War II

The *number* of US motor vehicle fatalities has declined since 1970 though unsteadily since fatalities are also strongly pro-cyclical, rising and falling in tandem with the economy. Motor vehicle fatality *rates* per vehicle miles traveled, by contrast, have declined more steadily from World War II until COVID-19, remaining somewhat pro-cyclical in that decline has been fastest before and during economic recessions. Exhibit 7 displays US motor vehicle fatalities (in red) and fatality rates (in green) from 1950 to 2018. Both fatalities and fatality rates trend mostly downward over time, fatality rates more smoothly and throughout than actual numbers of fatalities that rise until 1970 and then decline roller-coaster style with the ups and downs of the US economy. Ten recessionary periods are indicated by the gray vertical bars in Exhibit 7. As can be seen by comparing the gray bars with the white spaces in Exhibit 7, motor vehicle fatality rates (per 100 million miles driven) decline more sharply in recessions than in adjacent years for eight of ten recessionary periods (the exceptions are the January–July 1980 and the March–November 2001 recessions). Actual fatalities also decline more sharply in recessions than in adjacent years for the same eight of the ten recessions. The sharp increase in motor vehicle fatality rates following COVID-19, then, seems inconsistent with the downward trajectory of fatality rates during the majority of recessions occurring from 1950 to 2018.

**Exhibit 7:**
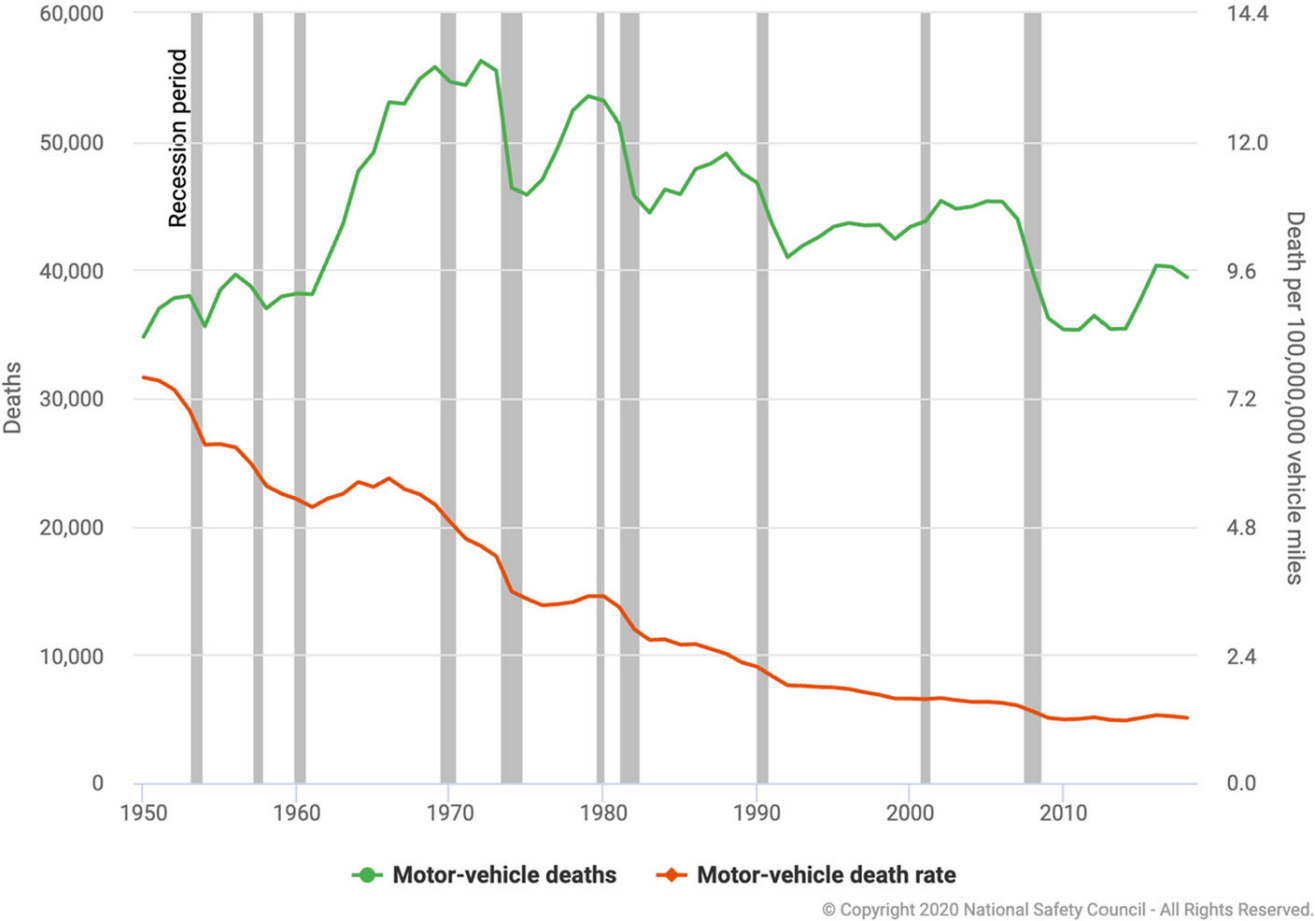
Historical motor vehicle deaths and death rates 1950–2018. Horizontal Bars Represent Recessions. Source: National Safety Council 2020d

To the best of my knowledge, no econometric study has identified the underlying causes of US motor vehicle fatalities or fatality rates from the 1950s until now. However, one study identified a unique factor associated with the rise and fall of motor vehicle fatalities from 2001 to 2011 (He 2016), the 11 years surrounding the Great 2008–2009 Recession that, prior to COVID-19, was the largest economic shock the USA suffered since the Great Depression of the 1930s. Of eight factors potentially associated with motor vehicle fatality rates across the 50 states, only the unemployment rate proved statistically significant: for each percentage point increase in the unemployment rate, the motor vehicle fatality rate decreased 2.82%. This result is attributable to decreased commercial activity during recessions, which removed a greater percentage of large trucks than passenger vehicles from the road and by inference reduced the lethality of traffic accidents. In months following COV1D-19 stay-at-home and business shutdown orders, as we have seen, total vehicle miles dropped 18–19% (U.S. Federal Highway Administration 2020c) while truck mileage decreased 20% (Teletrac Navman), a small difference, yet the number of trucks caught speeding, cornering harshly, and running stop signs actually increased. It seems unlikely, then, that the proportion of truck-involved accidents or their lethality changed greatly following COVID-19.

A second econometric study focused on vehicle miles traveled—not fatalities—among a sample of State Farm’s insured in Ohio’s 88 counties from August 2009 to September 2013 (Maheshri and Winston 2016). The county-level unemployment rate strongly predicted travel: each percentage point increase in unemployment was associated with a 14% decrease of vehicle miles traveled. The decrease in travel was greatest for drivers under 30 years of age, drivers over 60, drivers of older vehicles, and drivers who had filed an accident claim. The argument is that *unemployment disproportionately removed the most dangerous drivers from the road*. The inference is that *highway safety improves during recessions due to shifts in the driving population toward stably employed people predisposed to drive carefully*. A question currently unanswerable is whether COVID-19 stay-at-home orders increased the proportion of the less stably employed and the less cautious in the driving population.

### Motor Vehicle Fatality Rates During World War II

US vehicular traffic dropped sharply during World War II as a consequence of gasoline rationing. Rationing was implemented to limit the use of rubber, which was entirely imported, rather than petroleum where the USA had ample domestic supplies. Gasoline rationing began in seventeen Eastern Seaboard states on May 12, 1942, and was extended nationwide on December 1. To further limit gasoline consumption and rubber usage, a nationwide “victory speed” limit of 35 miles per hour was also mandated in May 1942. Both rationing and the “victory speed” remained in place until VJ Day, August 14, 1945. Beyond these restrictive actions, a nationwide propaganda campaign discouraging unnecessary and unnecessarily exuberant driving or “joy riding” also began in 1942. Exhibit 8 shows a 1942 Dr. Seuss (Theodore Geisel) cartoon where caricatures of Hitler and Tojo are encouraging a US joy rider to step on the gas.

**Exhibit 8:**
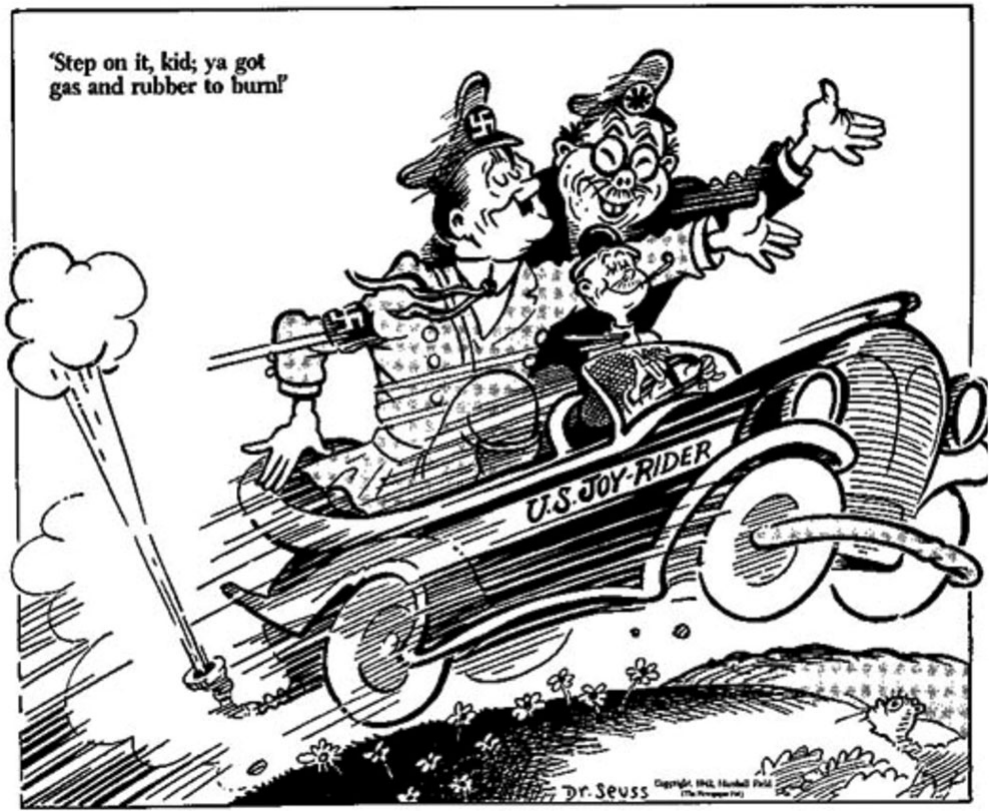
Giving the axis a lift. Source: National World War II Museum 2015

Wartime gasoline rationing reduced vehicle travel substantially from 1941 to 1945. The sharpest reductions were from 334 billion vehicle miles in 1941 to 268 billion miles in 1942 to 208 billion miles in 1943, a 21% drop from 1941 to 1942 and a further 26% drop from 1942 to 1943. Vehicle travel increased slightly from 1943 to 1944 and then rose substantially following VJ Day in 1945. Most interesting, however, is that aside from an upward spike in 1941 and a dip in 1942, the motor vehicle fatality rate was otherwise flat from 1939 to 1945. As shown in Exhibit 9, the vehicle fatality rate per 100 million miles ranged only from 10.71 to 10.92 from 1939 to 1942 except in 1941 when it was 11.43 and 1942 when it was 10.07.^2^ While the 1941–1942 decline in the motor vehicle fatality rate could be attributed to gasoline rationing, the 35 mile-per-hour “victory speed,” and the campaign against “joy riding,” the 1940–1941 and 1942–1943 increases in fatality rates remain inexplicable. One can only imagine what World War II motor vehicle fatality rates would have looked like had there been no “victory speed” or propaganda directed against unnecessary driving. Unknown is whether with so many fewer cars on the road these fatality rates would have spiked to much higher levels as happened following COVID-19 stay-at-home and business shutdown orders in 2020.

**Exhibit 9:**
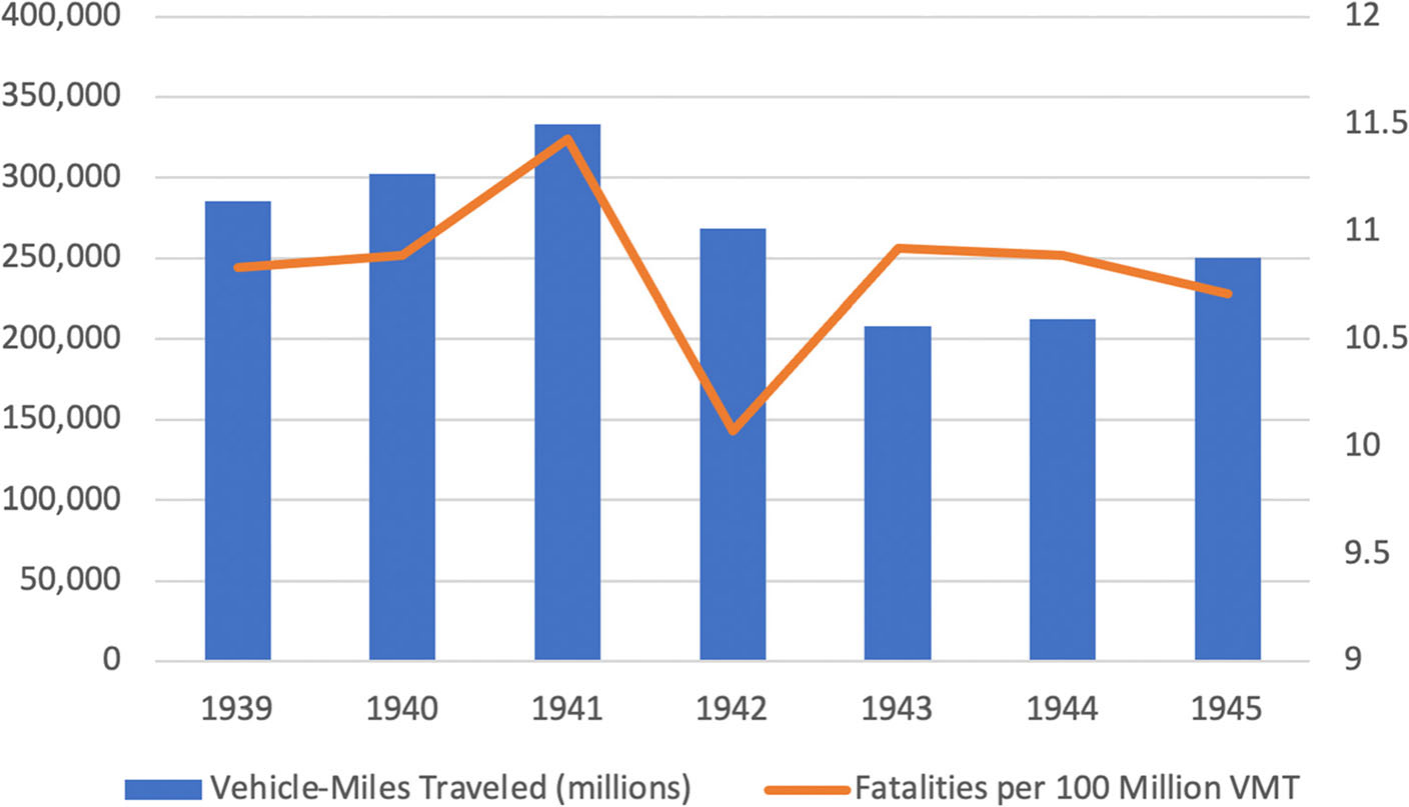
Annual vehicle miles traveled and fatalities per 100 million vehicle miles traveled (VMT), 1939–1945. Source: U.S. Federal Highway Administration 2020b

## What Are the Lessons?

In summary, they are five key empirically observed facts:

1. US vehicular traffic declined sharply in the wake of COVID-19 and then slowly recovered. Motor vehicle traffic was down 19% in March 2020 in comparison with March 2019 and down 37% in April, the first full month of COVID-19 lockdowns. The decline in traffic then abated to 26% in May, 13% in June, 11% in July, and 12% in August.
2. Motor vehicle fatality rates, measured as fatalities per 100 million miles of driving, increased sharply in comparison with 2019 and, perhaps more significantly, increased monthly from 1.24 in March to 1.48 in April, 1.56 in May, 1.63 in June before abating to 1.53 in July and returning to 1.60 in August. It appears, in other words, that from April 2020, there was a step-like discontinuity in motor vehicle fatality rates. Furthermore, the actual number of US motor vehicle fatalities per month exceeded 4000 in July 2020 for the first time since July 2007.
3. The percentage of injury accidents in New York City and of speeding violations in New York City and Massachusetts also increased during COVID-19, broadly consistent with the increase in motor vehicle fatality rates nationwide.
4. Unlike under COVID-19, vehicular traffic declined modestly in all post-World II recessions prior to COVID-19 and motor vehicle fatality rates declined in eight of these ten recessions.
5. Unlike under COVID-19, there was little change in motor vehicle fatality rates when World War II gasoline rationing coupled with a national 35 mile-per-hour speed limit and an anti-“joy riding” campaign strongly and effectively discouraged driving. Whether motor vehicle fatality rates have risen absent these stern measures is unknown.

### Modeling These Observations

The larger lessons depend on our framing of these empirical observations. The simplest model comes from traffic engineering where a focal problem has been the impact of highway congestion on accident rates. To oversimplify, traffic engineers fall into two camps, those finding that accidents increase in proportion to congestion (measured by traffic volume or the ratio of volume to road capacity) and those finding a curvilinear or U-shaped relationship where accident rates peak at extremely low and extremely high levels of congestion. Currently, the curvilinear camp is ascendant (Retallack and Ostendorf 2019).

As research studies accumulate more data points and, critically, measure congestion over shorter time intervals (in 1-hour intervals, for example, rather than in months), the more evidence tends toward the curvilinear or U-shaped model of the relationship of congestion to accidents. In curvilinear models where the horizontal axis is congestion, accidents rise steeply on the left side of the U driven by careless, inattentive driving and, of course, higher speeds. In Exhibit 10, I call the left side *Dodge City* where there is no sheriff. The right side is driven by the physical proximity of vehicles. I call this *Dodgem cars*, the amusement park ride where people strap themselves into small electric vehicles designed to bump each other. The labeling of the axes in Exhibit 10 follows the traffic engineering literature where the ordinate (*Y*-axis) is accidents and the abscissa (*X*-axis) is congestion. I’m treating these axes as analogs of fatality rates and total vehicle miles driven, inexact analogs to be sure but helpful in conveying the general points.

**Exhibit 10:**
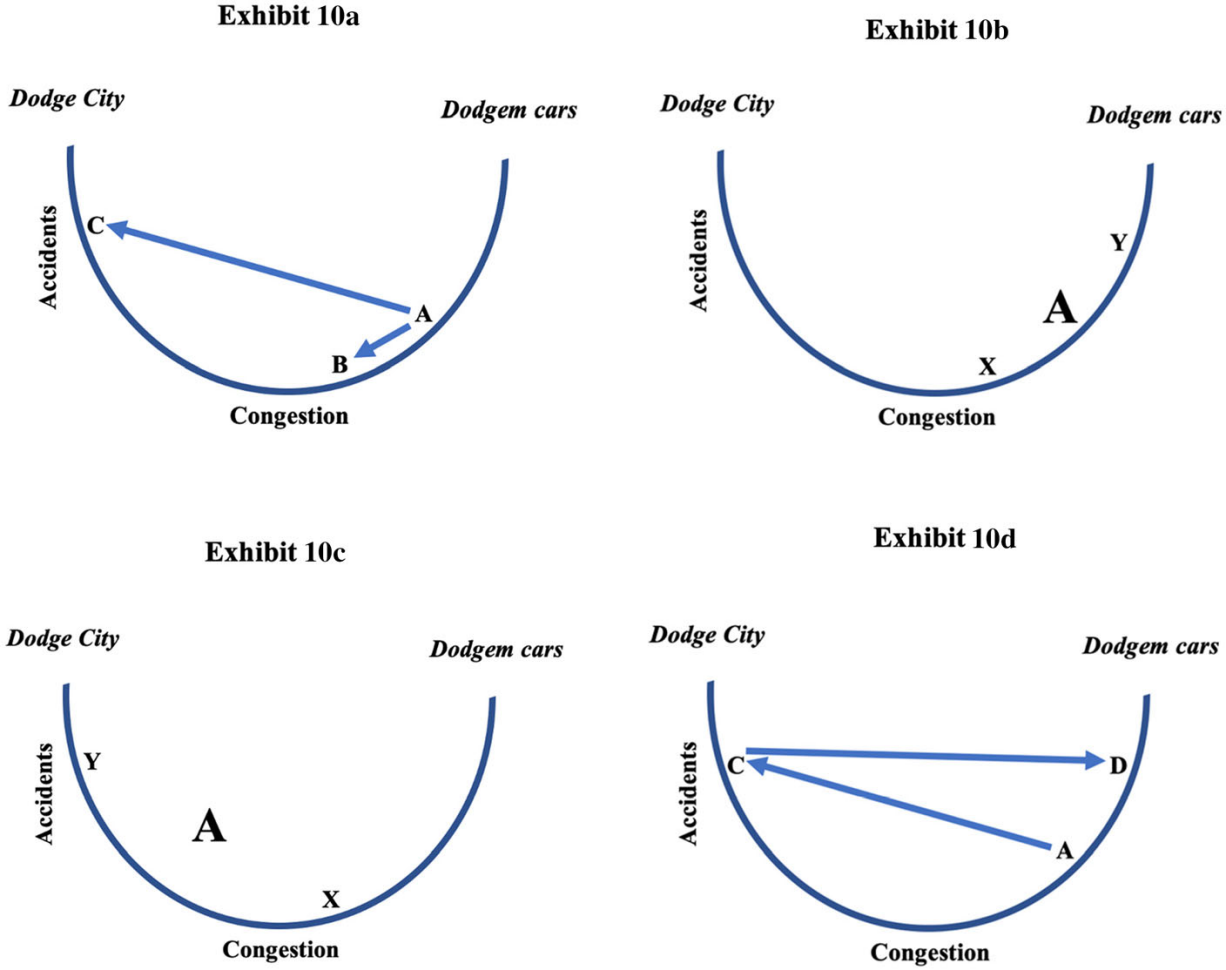
Four variations of the U-shaped congestion-accidents model. Congestion and accidents are treated as analogs of vehicle miles traveled and motor vehicle fatality rates. Exhibit 10a compares accidents following incremental (A➔B) versus sharp and discontinuous (A➔C) decreases in congestion. Exhibit 10b shows vehicles at different points (x, y) on the right of the congestion-accident curve and the impact of removing one type of vehicle. Exhibit 10c shows two populations of vehicles (x, y), observed congestion and accidents (A) if population difference is not recognized, and the impact of removing one population. Exhibit 10d describes how threshold effects sustain accidents despite decreasing congestion (C➔D)

In the simplest form of the curvilinear model (Exhibit 10a), point A represents “normal” accidents and congestion (or fatality rates and total vehicle miles or TVM). Point A is on the right side of the U where accidents increase with congestion (or fatality rates increase with TVM) though not linearly. Point B represents a modest reduction of congestion as in recessions. Along the path from A to B, accidents (fatality rates) decrease incrementally as congestion (TVM) also decreases incrementally. Along the path from A to C, accidents (fatality rates) increase incrementally as congestion (TVM) decreases dramatically moving from the right to the left side. The curvilinear accident-congestion model fits the observed precipitous decline in motor vehicle travel following COVID-19 stay-at-home orders and the concomitant increase in motor vehicle fatality rates.^3^

Exhibit 10b represents, albeit inexactly, econometric analysis of recessions and motor vehicle accidents and fatality rates. As in Exhibit 10a, point A represents “normal” accidents (fatality rates) and congestion (TVM). Imagine now that X represents passenger vehicles and Y trucks. Take the trucks off the road as in typical recessions, and point A moves to X—accidents (fatality rates) decline with congestion (TVM). Despite the imperfect analogy, the logic is straightforward: remove the most dangerous and highest mileage vehicles from the road, and accidents (fatality rates) will decrease with congestion (TVM). Or remove the most dangerous drivers from the road and accidents (fatality rates) will decrease with congestion (TVM).

We now extend econometric work on the connection of recessions to accidents and motor vehicle fatality rates to a scenario not yet considered: unobserved heterogeneity. This means differences between populations of drivers we do not or cannot observe. In Exhibit 10c, point A representing normal accidents (fatality rates) and congestion (TVM), though off the curve, is observed, while points X and Y though on the curve are not. Imagine X and Y are different populations of drivers driving on different roads and never mingling. Imagine also that the X population is slightly to the right, on the Dodgem car side of the curve, while Y is substantially to the left, on the Dodge City side. Again, neither X nor Y is observable but A, which combines X and Y and lies between X and Y, is. Imagine, further, that the X population limits its driving severely due to stay-at-home orders while the Y population continues to drive as usual. The consequence is that observable point A moves toward Y—in other words, accident rates (fatalities) appear to rise while congestion (TVM) appears to fall. However, no one’s risk of dying on the highways has changed. Rather, Y has become a larger portion if not the entirety of A. Since we were not able to observe the difference between X and Y and the changed composition of A, we infer falsely that A has moved toward Dodge City.

One form of heterogeneity of particular concern here is the rural-urban gap in motor vehicle fatality rates that in both 2017 and 2018 was 2:1 (U.S. Federal Highway Administration 2019). From Exhibit 3, we know, for example, that August 2020 rural motor vehicle miles recovered to 90% of August 2019 miles while urban vehicle miles recovered to 86%. The increase in the share of rural vehicle miles may have caused a small increase in the overall motor vehicle fatality rate, but by how much is uncertain given that the urban fatality rate also increased. Another potential source of heterogeneity is in the caution exercised by drivers. It is possible that less cautious drivers and drivers having urgent business remained on the highways despite stay-at-home and shutdown orders while more cautious drivers and those having less urgent business and business that could be conducted at home actually stayed home.

In Exhibit 10d, the A➔C path from Exhibit 10a remains but is a one-way street: there is no C➔A return path as congestion (TVM) returns. Rather, the return path, the Hobbesian scenario, is a horizontal line, C➔D, where D is a “new normal” frequency of accidents (fatalities) well above A and insensitive, as shown, or less sensitive to congestion, as could be shown in a shallower U, than the A➔C path. Hence, Exhibit 10d draws attention to a critical and at this point unanswerable question: has COVID-19 caused motor vehicle fatality rates to ratchet upwards such that they will remain permanently elevated?

Ratcheting may have occurred. The explanation lies in a common thread in the literature on small group behavior, the behavior of mobs, and the adoption of innovations: threshold models of contagion (Asch 1956; Granovetter 1978; Watts 2004, especially ch. 8, “Thresholds, Cascades, and Predictability”).

Let me sketch how threshold models work in the context of riots. I am a peaceable person and disinclined to join a riot because I generally do not like rioters and do not relish the prospect of spending time in jail—in other words, my rioting threshold is quite high. Still, if my friends have joined the riot and the riot has become so large than the likelihood of arrest is low, I may join the riot because the opportunity to join my friends and snatch a piece of jewelry outweighs my distaste for other members of the mob and disinclination to jail time.

I’ll now apply the threshold model to the highways. Assume we are at point C in Exhibit 10d and I resume driving. Once I’m on the highway I find almost everyone doing 75 miles per hour even though the speed limit is 55 and I would normally drive at 60. Rather than obstructing traffic and enduring honking horns and clenched fists, I speed up. So do other drivers as they gradually return to the road. At each step of the process, drivers with higher thresholds of speeding become speeders since every larger numbers of fellow drivers are speeding. Average speed stays up and accident and fatality rates remain elevated as traffic returns to normal. The prediction, consistent with the model displayed in Exhibit 10d and the July National Safety Council data, is that neither accidents nor motor vehicle fatality rates will decrease as congestion or vehicle miles traveled return toward normal with the opening of the economy. The U-shaped model from traffic engineering, then, morphs into something more closely resembling a Kryptonite New York bicycle lock, a “new-U” with a bar across the top.

### Hobbesian Highways: what We Can and Cannot Do About Them

Though it’s hard to prove, the data are highly suggestive of Hobbesian highways following COVID-19. Initially, motor vehicle fatality rates inched up as vehicular traffic dropped precipitously. This was an unusual occurrence as fatality rates normally decline when the economy slows and traffic thins. Subsequently, fatality rates continued to rise as traffic returned. The impression is that we retreated from norms of everyday driving or, at a minimum, shifted these norms in response to COVID-19 and stay-at-home and business shutdown orders. When we step back from mileage and fatality statistics and think about what ultimately caused fatality and injury accident rates to spike, the initial reaction might be that incivility has sprouted everywhere. Perhaps so, but the story of motor vehicle fatalities allows us to specify some of the mechanisms through which incivility propagates. In a sentence: the emptying of the highways in late March and April gave license to the remaining drivers to disregard the rules of the road such that others did the same when the others resumed driving from May through August. While not precisely Hobbes’s state of nature, driving and dying on the highways has become a little like “ . . . that to be every mans that he can get; and for so long, as he can keep it . . .”

Is there a solution, even a Hobbesian solution, to what appear to be Hobbesian highways? Highway safety has not been an intractable problem in the past. The federal government and advocacy groups have promoted highway safety since President Harry Truman convened a national highway safety conference in 1946. These efforts have yielded considerable success—compare World War II era motor vehicle fatality rates in the vicinity of 11 per 100 million vehicle miles with the pre-COVID-19 rate of 1.15. Still, the challenges posed by COVID-19 morbidity and mortality, rising gun violence and domestic violence, and racial disparities leave little space for attention to motor vehicle fatality *rates* when the actual numbers of fatalities, so far, have not increased *much*. Nonetheless, they have increased: actual motor vehicle fatalities in July and August were the highest in 13 years.

Still, this glimpse into the impact of COVID-19 on highway fatality rates should help reshape our response to the pandemic. What happened on the highways was unexpected, without precedent, and warns us, as Hobbes does, that our social fabric is always at risk. We have two competing—and, unfortunately, politically charged—narratives about COVID-19. One is the public health narrative, the other the economic narrative, the former focused on containing the coronavirus, the latter on maintaining jobs. So far, we have not had a reckoning of the social consequences of the actions avoided and the actions belatedly taken in response to COVID-19, and we probably will not for a while. If the analysis here is correct, which is not guaranteed, and if our roads and highways are microcosms of society and have inched toward Hobbes’s state of nature, then our concerns must go beyond eradicating COVID-19 and rebuilding our economy. We will also need to find ways to reverse the unanticipated and untoward effects of having so greatly distanced ourselves from each other.

## Footnotes

1 These risks are algebraically related: population risk [motor vehicle fatalities/population] = motor vehicle fatality rate [motor vehicle fatalities/motor vehicle miles driven] * driving rate [motor vehicle miles driven/population]. This is an identity. It is not causal and does not mean that fatality rates will automatically drop when driving increases.

2 Motor fatality rates in the 1940s were 7–8 times higher than today. Note also that the vertical axis of Exhibit is annual rather than monthly vehicle miles traveled as in Exhibit 4.

3 Note that the famous “Peltzman effect,” which claims that the safer drivers feel, the more dangerously they will drive (and hence, for example, automotive safety systems have little or no net effect on accidents and injuries) is a special case of the curvilinear traffic engineering model. Drivers at point C will feel safer than at point A because there are many fewer cars on the road. However, they are actually at greater risk due to speeding or inattentiveness caused by overconfidence, consistent with the “Peltzman effect.” By contrast, drivers at point B will feel somewhat safer than at point A and will actually be somewhat safer because there is more space between cars yet not so much space that they are overconfident (Peltzman 1975).
